# A composite docking approach for the identification and characterization of ectosteric inhibitors of cathepsin K

**DOI:** 10.1371/journal.pone.0186869

**Published:** 2017-10-31

**Authors:** Simon Law, Preety Panwar, Jody Li, Adeleke H. Aguda, Andrew Jamroz, Rafael V. C. Guido, Dieter Brömme

**Affiliations:** 1 Department of Biochemistry and Molecular Biology, Faculty of Medicine, University of British Columbia, Vancouver, British Columbia, Canada; 2 Centre for Blood Research, University of British Columbia, Vancouver, British Columbia, Canada; 3 Department of Oral Biological and Medical Sciences, Faculty of Dentistry, University of British Columbia, Vancouver, British Columbia, Canada; 4 Centro de Inovação em Biodiversidade e Fármacos, Instituto de Física de São Carlos, Universidade de São Paulo, São Carlos, Brazil; Stanford University, UNITED STATES

## Abstract

Cathepsin K (CatK) is a cysteine protease that plays an important role in mammalian intra- and extracellular protein turnover and is known for its unique and potent collagenase activity. Through studies on the mechanism of its collagenase activity, selective ectosteric sites were identified that are remote from the active site. Inhibitors targeting these ectosteric sites are collagenase selective and do not interfere with other proteolytic activities of the enzyme. Potential ectosteric inhibitors were identified using a computational approach to screen the druggable subset of and the entire 281,987 compounds comprising Chemical Repository library of the National Cancer Institute-Developmental Therapeutics Program (NCI-DTP). Compounds were scored based on their affinity for the ectosteric site. Here we compared the scores of three individual molecular docking methods with that of a composite score of all three methods together. The composite docking method was up to five-fold more effective at identifying potent collagenase inhibitors (IC_50_ < 20 μM) than the individual methods. Of 160 top compounds tested in enzymatic assays, 28 compounds revealed blocking of the collagenase activity of CatK at 100 μM. Two compounds exhibited IC_50_ values below 5 μM corresponding to a molar protease:inhibitor concentration of <1:12. Both compounds were subsequently tested in osteoclast bone resorption assays where the most potent inhibitor, 10-[2-[bis(2-hydroxyethyl)amino]ethyl]-7,8-diethylbenzo[g]pteridine-2,4-dione, (NSC-374902), displayed an inhibition of bone resorption with an IC_50_-value of approximately 300 nM and no cell toxicity effects.

## Introduction

Thiol-dependent cathepsins are found in all life forms and have a vital role in mammalian intra- and extracellular protein turnover [1]. They are members of the papain-like family (CA clan, C1 family) and have 11 proteases encoded in the human genome (cathepsins B, C, F H, K L, O, S, V, W and X). In particular, cathepsins K, S, and V are potent elastases with cathepsin K (CatK) also being a highly effective and unique collagenase capable of cleaving at multiple sites within triple helical collagens [2–4]. These proteases have been implicated and targeted in various musculoskeletal and cardiovascular diseases [5–7].

Major efforts have been undertaken to develop potent cathepsin inhibitors [8–10]. However, all compounds in development are active site-directed inhibitors, which completely block the activity of the enzyme. Because cathepsins are multifunctional proteases, it is likely that blocking their entire proteolytic activity will have unwanted side effects [11]. This may explain in part the failing of clinical trials of CatK inhibitors for the treatment of osteoporosis. Patients experienced scleroderma-like phenotypes and revealed increased risks in cardiovascular events such as stroke despite showing excellent bone-preservation outcomes [12–14].

Our previous studies have demonstrated that the degradation of extracellular matrix (ECM) proteins such as collagens and elastin requires specific exosite binding sites. These sites are needed for the formation of protease oligomers in the presence of glycosaminoglycans in the case of CatK-mediated collagenase degradation [15, 16]. Blocking protein-protein, protein-glycosaminoglycan, or specific substrate binding sites with small molecules will allow the selective inhibition of the collagenase and elastase activities of cathepsins without affecting their active site and thus the hydrolysis of non-ECM substrates. We termed these sites ectosteric sites to differentiate them from allosteric sites as they do not affect the catalytic site upon inhibitor binding. Ectosteric inhibitors targeting these sites will thus represent substrate specific inhibitors, which selectively can block the disease-relevant activities of cathepsins. We have recently demonstrated that the selective inhibition of the enzyme’s collagenase activity in osteoclast bone-resorption assays and in an osteoporosis mouse model can be achieved without blocking its TGF-ß1 degrading activity correlated to some of the side effects seen in CatK inhibitor clinical trials [17, 18].

In this study, we adopted the library docking method with the aim to identify novel scaffolds for ectosteric substrate-specific CatK inhibitors. Potential inhibitors for CatK-mediated collagen degradation were identified using a computational approach involving multiple docking algorithms. We identified four common chemical scaffolds and several other compounds that may be used as a starting point for further development. Of 160 compounds identified from the NCI-DTP repository and tested in enzymatic assays, 28 compounds effectively blocked the collagenase activity without disrupting the active site activity in CatK. Two of these compounds were active at about a 12-fold molar excess over CatK and revealed potent antiresorptive activity in osteoclast bone degradation assay.

## Materials and methods

### Molecular docking of NCI/DTP chemical repository library to ectosteric site 1

Chemical structure data of the NCI/DTP Chemical Repository was downloaded from PubChem for molecular docking analysis. The preliminary study subset of compounds was selected using BioActive and rule of 5 filters, leaving 14,045 compounds. The appropriate three-dimensional structures were generated using LigPrep and OPLS3 force fields and ionization states generated at pH 5.5. (Schrödinger Inc.) [19]. Geometric rotamers generated for each compound was limited to twelve and three per ligand for the preliminary and complete library studies, respectively, and were exported as SDF files. The enzyme molecule used for docking was an inhibitor-bound CatK (PDBID: 1ATK) with the inhibitor and heteroatoms removed; an inhibitor-free CatK structure was unavailable at the time. Additional processing of the enzyme molecule was performed in the respective programs prior to docking.

### Surflex docking

Docking and similarity calculations were carried out using standard protocols with Surflex-Dock Geometric (SFXC) as part of the Sybyl-X Suite [20]. The protein structure was prepared using the Sybyl-X protein preparation wizard with hydrogen atoms added and side chains rotamers corrected. We generated the protomol used for docking by exploiting the residue mode (Ser95) with a threshold of 0.5 and a bloat of 4. The prepared ligand sets were then docked to the protomol using the default settings with protein hydrogen movement and CScore calculations enabled. Poses were sorted by their CScore calculations and exported for visualization into PyMOL. For the reevaluation of the docking of compounds **1** and **3**, maximum conformations per fragment were increased from 20 to 50 and the search density was increased ten-fold from 6 to 60.

### Glide docking

Glide docking was performed using Glide docking suite (Schrödinger Inc.) with the previously prepared ligand set [21]. The protein was prepared using the protein preparation wizard with the standard configuration, where hydrogens were added and the overall structure was refined with its free energy minimized. The receptor grid was generated using Ser95 as the centroid for the binding site with a binding box of 10 Å in the putative ectosteric site. No additional constraints were specified and the settings were left to their default values. The prepared ligands were docked using the aforementioned receptor grid using SP (standard precision). Ligand sampling was allowed to be flexible and other settings were left at the default values. Post-dock minimization was performed and a maximum of five poses were written for each compound. Poses were sorted by their Glide score and exported for visualization into PyMOL. For the reevaluation of the docking of compounds **1** and **3**, extra precision docking was used (XP mode) with a maximum of 100 poses used for post-docking minimization. Physicochemical properties including cLogP and cell membrane permeabilities were determined using QikProp (Schrödinger Inc.) for compounds **1** and **3**.

### GOLD docking

GOLD docking was performed using the GOLD Suite (Hermes 1.7.0) with the prepared ligand set [22]. Protein preparation was performed in GOLD where hydrogens were added and side chains rotatable bonds were fixed. The binding site was defined using Ser95 as the center with a surrounding box of 10 Å. Cavity detection and docking were performed using the default GOLD settings with ligand flexibility and rescoring enabled. Poses were ranked by the CHEMPLP scores and exported for visualization into PyMOL. For the reevaluation of compounds **1** and **3**, search efficiency was increased from 25% to 200% and all solutions were kept for evaluation.

### Collagenase and gelatinase assays

The collagenase inhibitory activities of all identified compounds were measured using a collagen degradation assay. Soluble bovine type I collagen (0.6 mg/mL) was incubated with 400 nM recombinant human CatK, in the presence or absence of 200 nM chondroitin 4-sulfate (C4-S) in 100 mM sodium acetate buffer, pH 5.5, containing 2.5 mM dithiothreitol (DTT) (Sigma-Aldrich Canada, Oakville, Ontario, Canada) and 2.5 mM ethylenediametetraacetate (EDTA) (Sigma-Aldrich). In the experiments involving detergent to minimize inhibitor aggregation, 0.001% or 0.005% Triton X-100 was added to the buffer with the inhibitor. The recombinant human CatK was expressed in *Pichia pastoris* and purified as previously described [23]. Selected compounds identified by molecular docking were ordered from the National Cancer Institute through the Developmental Therapeutics Program (Rockville, MD). All compounds were dissolved in DMSO as a 20 or 10 μM stock. To minimize the effect of solvent on the activity of CatK, all reactions were kept below 1% DMSO, where no solvent effect was observed. After incubation at 28°C for 4 hours, 1 μM of E-64 was added to block the residual activity of CatK.

Digestion with MMP-1 was performed in 50 mM Tris-HCl, pH 7.4, containing 200 mM NaCl and 5 mM CaCl_2_ at 28°C for 4 h. Reactions were stopped by adding 25 mM EDTA as previously described [24]. Samples were analyzed using 10% SDS-PAGE gels and stained with Coomassie. The resulting bands of α-collagen chains were quantitatively assessed using ImageJ (Version 1.5) [25] and IC_50_ graphs were plotted using GraphPad Prism (GraphPad Software, Version 5.0, La Jolla California USA).

Gelatin (0.6 mg/mL) degradation assays were performed in the same manner as the collagen degradation experiments with 10 nM enzyme and were incubated at 37°C for one hour at the appropriate pH values for the individual proteases (CatK, pH 5.5; MMP-1, pH 7.4; trypsin, pH 8.8).

### Z-Phe-Arg-MCA cleavage assays

Evaluation of a potential active site inhibition of the compounds was performed using the fluorogenic Benzyloxycarbonyl-Phe-Arg-7-amido-4-methylcoumarin (Z-Phe-Arg-MCA) substrate (Bachem Americas, Inc, Torrance, California, USA) as previously described [26]. The enzymatic activity of CatK was monitored by measuring the rate of release of the fluorogenic group, amino-methyl coumarin at an excitation wavelength of 380 nm and an emission wavelength of 450 nm using a Molecular Devices SpectraMax Gemini spectrofluorometer. Inhibitors were added prior to measurement of enzyme activity. The assays were performed at 25°C at a fixed enzyme concentrations (5 nM) and substrate concentration (5 μM) in 100 mM sodium acetate buffer, pH 5.5, containing 2.5 mM DTT and 2.5 mM EDTA. Z-Phe-Arg-MCA hydrolysis with trypsin was carried out in 50 mM Tris-HCl, pH 8.8 at 10 nM enzyme concentration.

### Human osteoclast cultures and bone resorption analysis

Osteoclasts were generated from mononuclear cells isolated from human bone marrow tissue (Lonza, Walkersville, MD). The bone marrow cells were centrifuged at 400 g for 5 min and the pellet was re-suspended in 10 mL α-MEM (α-Minimal Essential Media) and layered on 10 ml Ficoll-Plaque media solution. After centrifugation at 500 g for 30 min, the white interface containing the monocytes was harvested and washed twice with α-MEM. Cells were cultured in α-MEM containing 10% FBS and 25 ng/mL M-CSF for 24 hours and then cultured in 25 ng/mL RANKL and 25 ng/mL M-CSF for 7 days. Differentiated osteoclasts (100,000 cells per slice) were then seeded on each bone slice (5.5 mm diameter and 0.4 mm thickness; self-made) in the presence or absence of inhibitors and incubated for 72 hours at 5% CO_2_ and 37°C with the DMSO concentration at 0.1%. The inhibitor concentration range tested varied between 50 nM to 3 μM.

To compare the effects of the compounds on cell survival, the metabolic activity of osteoclasts was determined using the CellTiter-Blue Viability Assay (Promega, Madison, WI, USA). Bone slices from each condition (inhibitor-treated and control groups) were fixed in 4% formaldehyde and subsequently stained for tartrate-resistant acid phosphatase (TRAcP) activity (Acid Phosphatase, Leukocyte (TRAP) Kit; Sigma-Aldrich). Aliquots from cell culture media were used to determine the CTx-I concentration (MyBiosource ELISA kit, San Diego, CA). CTx-I is a CatK-specific C-terminal cleavage product of triple helical type I collagen. The total number of osteoclasts per bone slice was determined after TRAcP staining. Cells with ≥2 nuclei were counted as osteoclasts. After 72 h, bone slices from each condition were incubated in filtered water for cell lysis and cells were removed using a cotton stick. Subsequently the resorption cavities were stained with toluidine blue and observed by light microscopy. The number of resorption events and eroded bone surface area were determined as previously described [27]. All light microscopic analyses were performed using a Nikon Eclipse LV100 microscope and a Nikon Eclipse Ci microscope.

## Results

### Identification and characterization of ectosteric site 1

**Fig 1:**
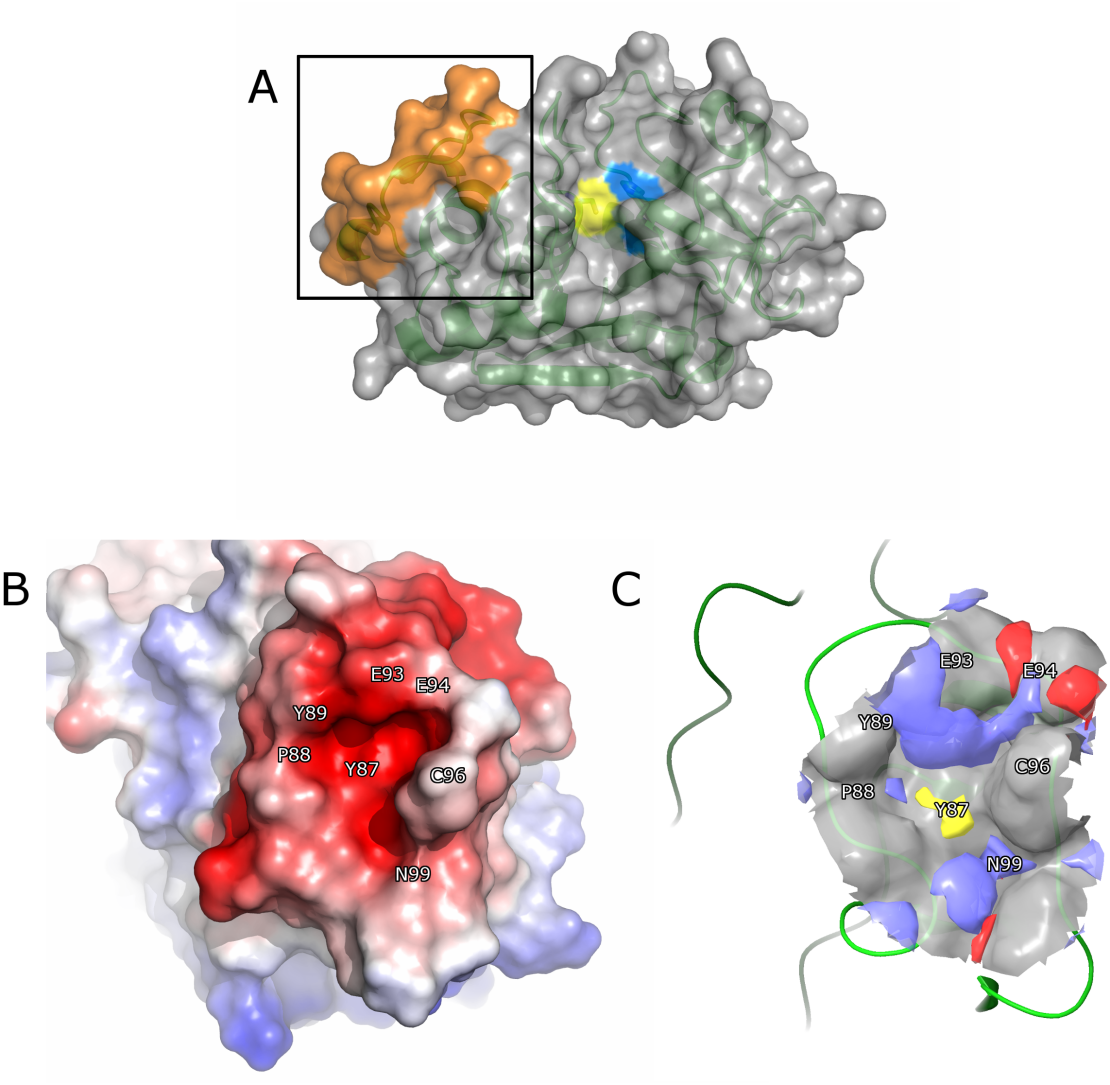
Binding site analysis of ectosteric site 1 in CatK. **(A)** Overview of CatK (PDB ID: 1ATK) shown in surface and ribbon form. The active site residues, Cys25 and His162, are colored in yellow and blue, respectively. Ectosteric site 1 is highlighted in the box and colored in orange. **(B)** The electrostatic potential surface of ectosteric site 1 in CatK displays electronegativity throughout the protein-protein interface as a result of negatively charged residues in this region. **(C)** The binding pocket in ectosteric site 1 displayed high theoretical druggability (Sitemap, Schrödinger Inc.), (druggability score: 0.816; druggability threshold: 0.80; site score: 0.715) as a result of its favorable geometry and size. Most of the area surrounding the cavity is hydrophilic (red for hydrogen bond-accepting sites, blue for hydrogen bond-donating sites). The potential interacting residues are located surrounding the binding site, Glu93, Glu94, and Gln99 and are shown in blue. The hydrophobic residue, Tyr87, can be seen in the center of the binding site and is shown in yellow.

Previous studies of the mechanism of the collagenase activity in CatK have implicated ectosteric site 1 as a site required for efficient collagen cleavage [16, 28]. Ectosteric site 1 is located on the surface of the L-domain of CatK and represents a protein-protein interaction site required for the formation of collagenolytically active dimers [28]. The site consists of a well-defined cavity and is part of a surface loop consisting of residues 84–100 (Fig 1A). In order to identify inhibitors binding in ectosteric site 1, a molecular docking approach was used. We first investigated the druggability of this site by analyzing the molecular surface and performing computational analysis using Sitemap (Schrödinger Inc.).

The binding pocket of ectosteric site 1 consists mostly of negatively charged surface residues with Tyr87 forming the hydrophobic interior of the pocket and residues Glu93 and Glu94 serving as potential hydrogen bond interacting partners with compounds binding in the pocket (Fig 1B). Residues 95 to 100 form the outer rim of the binding site and mostly consist of neutral residues. Other potential interacting residues including Gln92 and Glu84 are found near the exterior of the binding pocket but may play a role in determining the binding of larger compounds that occupy space outside the defined pocket.

The binding site was further evaluated using Sitemap, which characterizes the surface properties of the protein to determine its druggability using parameters such as its cavity size, exposure to solvent, hydrogen bond acceptors and donors as well as its hydrophobicity and hydrophilicity [29]. Sitemap analysis of the binding site revealed a favorable site score of 0.935 and a druggability score of 0.816 (druggability threshold = 0.80) [29]. The analysis also revealed hydrogen bond-donating regions surrounding the surface of the binding site as predicted by the amino acid sequence as well as a hydrophobic interior consisting of residue Tyr87 (Fig 1C). Hydrogen bond-accepting regions were found towards the periphery of the site. Based on these results, ectosteric site 1 was shown to be a druggable binding site and compounds that strongly interact with this binding site are likely to be small molecules with hydrophobic characteristics. These compounds would also interact with the hydrogen bond donor and acceptor residues lining the pocket.

### Screening of druggable subset library using composite docking method

**Fig 2:**
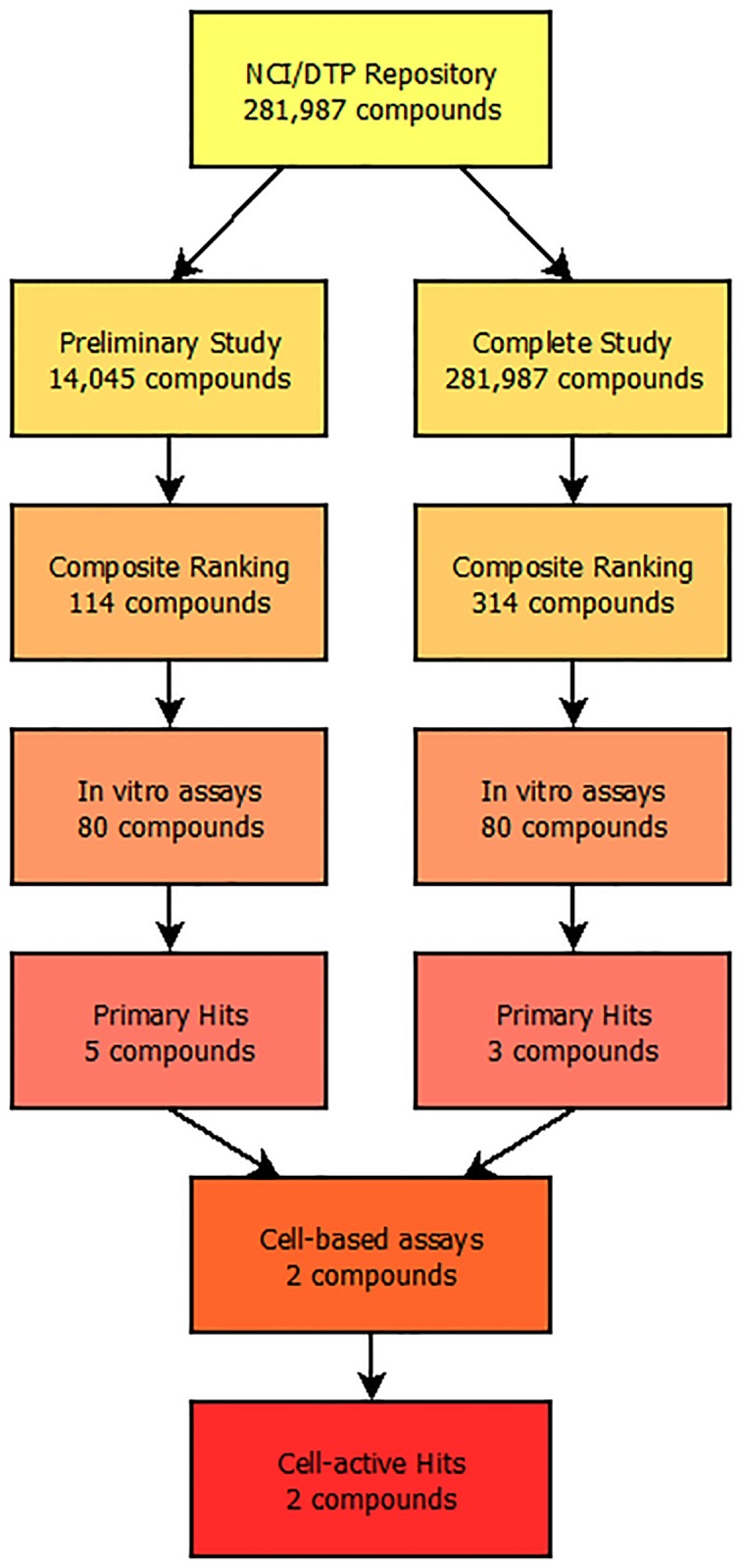
Screening and evaluation workflow for the identification of ectosteric site 1 inhibitors of CatK. The screening procedure used to identify potential collagenase inhibitors of CatK from the NCI/DTP Repository. A composite virtual screening method involving GOLD, Glide, and Surflex was used to screen and identify potential hits and a total of 160 compounds were tested in *in vitro* assays. Eight compounds were identified as potent collagenase inhibitors with IC_50_ values below 20 μM and two compounds were tested in osteoclast-based bone degradation assays with both inhibitors displaying bone resorption inhibition.

The NCI/DTP Chemical Repository containing pure synthetic compounds and natural products with diverse sets of chemical scaffolds comprises 281,987 small molecules ranging from 18 Da to 3,880 Da. This library of compounds was used as the ligand set for docking to ectosteric site 1 and the complete workflow can be found in Fig 2. Based on the size and pharmacological properties of the binding site determined in SiteMap, a subset of compounds was first selected using the compounds’ drug-like properties and bioactivity based on selection criteria using the Lipinski’s rules of five for initial testing [30]. These rules limit compounds to no more than five hydrogen bond donors and ten hydrogen bond acceptors with a molecular weight less than 500 Daltons for druggability. With these filters, the number of compounds tested was reduced to 14,045. Three-dimensional structures of the ligands were generated and prepared using LigPrep in Maestro (Schrödinger Inc.) prior to screening. OPLS3 force fields were generated with the possible ionic states limited between pH 5.5 ± 2.0 due to the pH activity profile of CatK and the pH of the *in vitro* experiments. Ligand flexibility was taken into account and up to 12 conformers were generated for each compound. The resulting prepared ligand sets were used for all three screening methods.

To overcome some of the weaknesses associated with computer screening approaches using a single algorithm, we chose to employ a composite docking method involving three separate algorithms, Surflex, Glide, and GOLD. Each docking algorithm uses its own method in the configuration of the binding site. For Surflex, a protomol (binding site) of ectosteric site 1 was generated using residues 88 to 92 with a bloat value of 4. The resulting protomol encompassed the entire binding site evaluated using SiteMap and was representative of ectosteric site 1. Both Glide and GOLD used a receptor grid for defining the binding site and a 10 Å box was generated using Ser95 as the centroid site. Both of these grids covered the entire volume occupied by ectosteric site 1 and the binding site identified in Sitemap.

Since the scoring algorithms are unique and address different binding parameter, we expected that compounds with simultaneous high scores in all three docking methods have a higher likelihood to be potential inhibitors. The top-ranked compounds from each individual docking method were combined and hits were defined as compounds with scores in the top 10% in all three methods. Of the 14,045 compounds docked to ectosteric site 1, 99 compounds fulfilled this inclusion parameter. Compounds were then ranked based on their composite score, which was the average of the ranking attained in each individual method.

**Fig 3:**
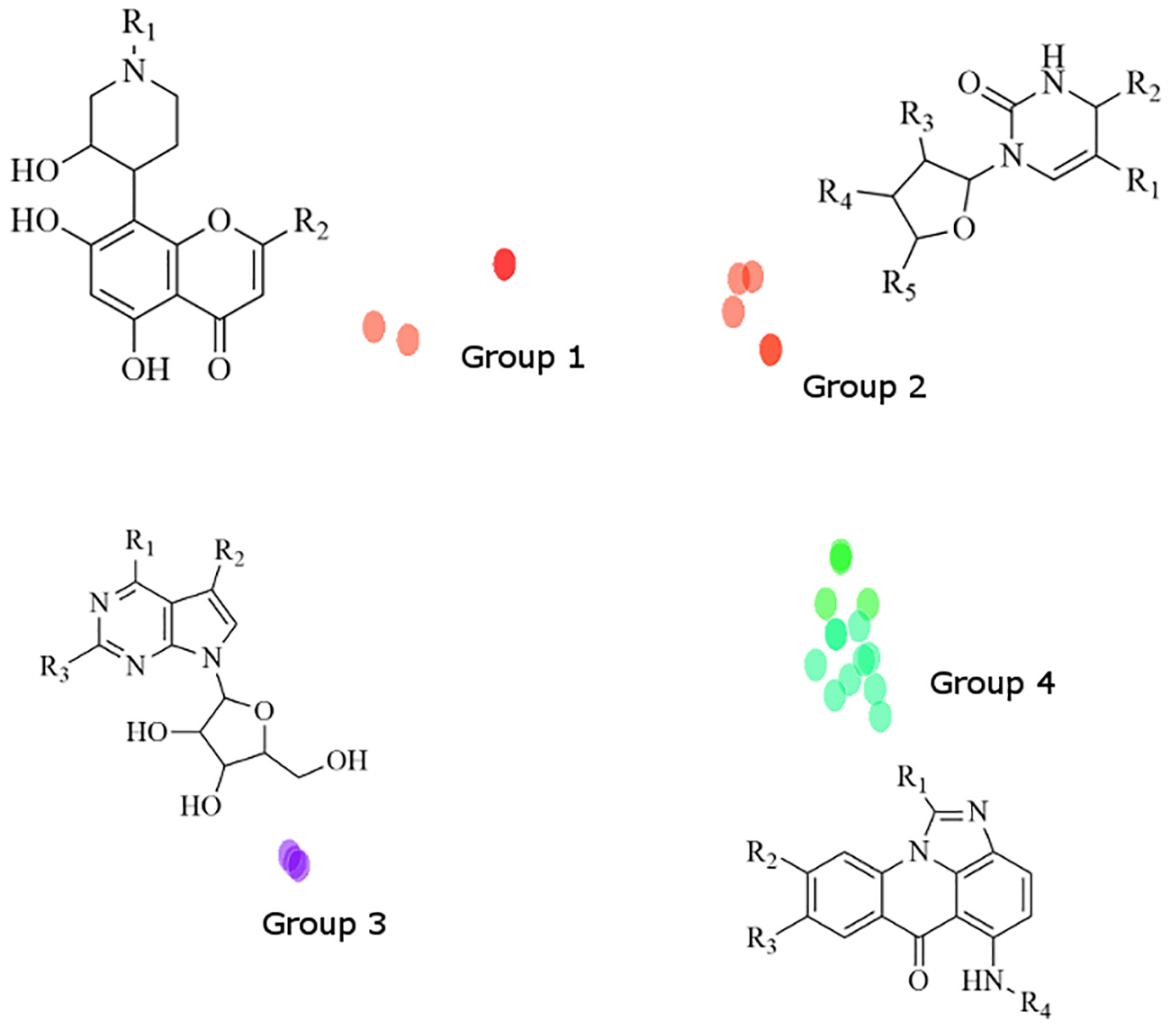
Chemical similarity mapping of hits identified through molecular docking. The composite method identified 25 compounds that could be grouped into four different scaffolds. Group 4 contained the highest number of compounds with 13 identified putative inhibitors. A complete list of compounds can be found in the Supplementary Information (S1 Table).

In order to visualize the chemical relatedness and potential scaffolds of the structures identified, the compounds were then clustered according to chemical similarity using their Tanimoto fingerprints in Sybyl-X (Fig 3) and an outlier cutoff of a minimum of 80% chemical similarity. The resulting structural similarity map revealed four groups of high chemical similarity containing a total of 26 compounds. The remaining 73 compounds did not meet the similarity cutoff and displayed different scaffolds. One particular cluster (Group 4) contained a family of 13 acridinone-related compounds with a common chemical scaffold that interacted favorably with ectosteric site 1 based on their predicted poses. Groups 1 to 3 contained compounds with more flexible scaffolds such as the purine-thione and chromanone-like structures that fit into the defined binding site of ectosteric site 1. The chemical scaffolds of all the families of compounds identified contained the potential interactions identified from the Sitemap analysis. These include a hydrophobic core and several hydrogen bond donators that can interact with the ectosteric site residues. The predicted poses for these compounds take advantage of these properties and display numerous interactions with these residues.

Since compounds with the same scaffold may also bind in a similar manner to the ectosteric site, a chemical similarity search was performed using scaffolds from the four high similarity clusters in the NCI-DTP database and yielded an additional 16 compounds, which were also considered for further *in vitro* testing. Since the compounds all scored in a similar range in the respective docking methods, further selection was performed based on the visual examination of the individual binding poses and the interactions the compounds made with the protein. Higher priority was placed on compounds with multiple hydrogen bonding interactions with the protein as well as those with substantial hydrophobic interactions with the core of the ectosteric site 1. Compounds with multiple conformers which all scored highly were also given greater consideration. From the total of 115 (99+16) compounds, we evaluated the activity of 80 compounds in collagenase and peptidase assays. Eleven compounds that were unavailable from the NCI/DTP Repository were not included.

**Fig 4:**
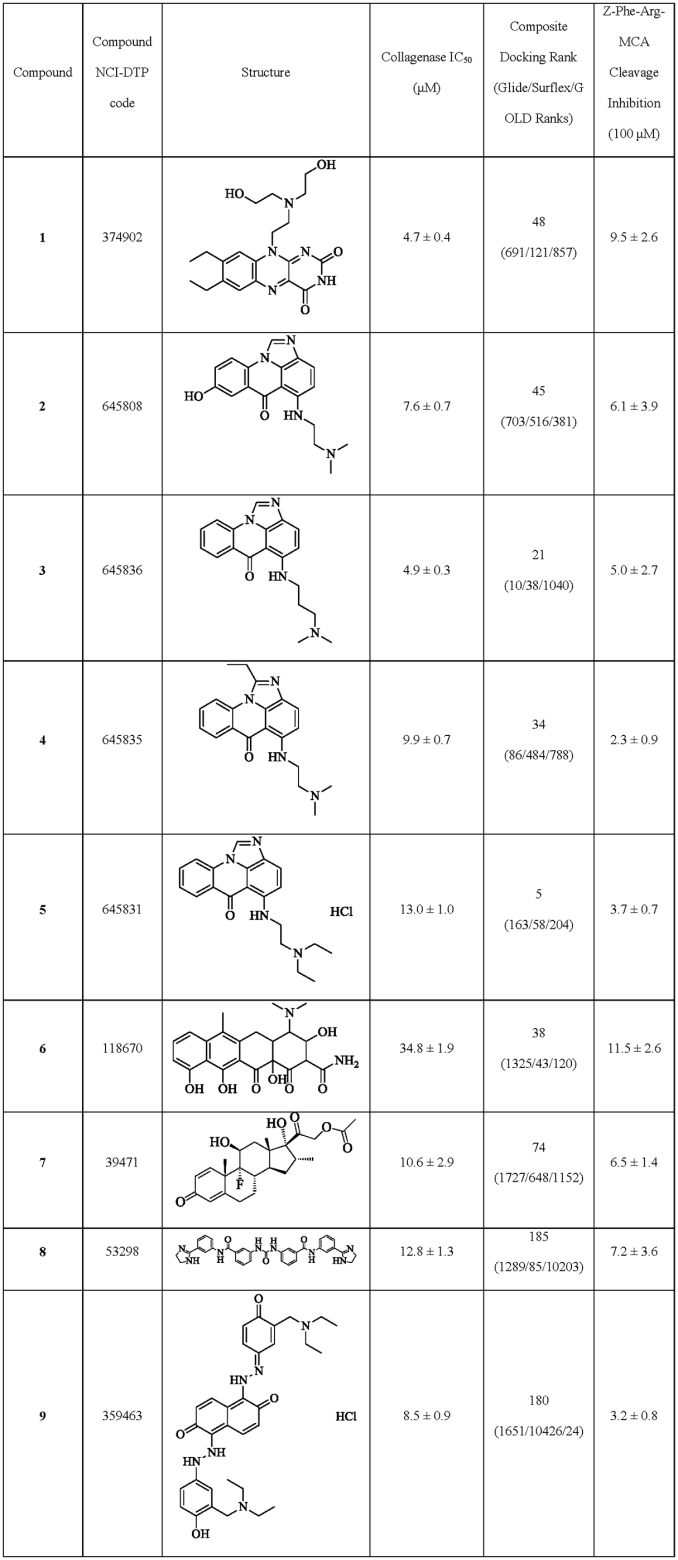
Summary of collagenase inhibitors identified through composite docking from the NCI-DTP repository.

To evaluate the efficacy of these compounds in inhibiting the collagenase activity of CatK and without blocking the active site of the protease, type I collagen degradation and Z-Phe-Arg-MCA hydrolysis assays were performed. Of the 80 compounds screened in the collagenase assay, 16 compounds showed inhibition at 100 μM and were further subjected to testing at lower concentrations (S2 Table). IC_50_-values were determined for the most potent compounds. Five of these compounds had IC_50_-values of below 20 μM for collagen degradation in the presence of 400 nM CatK enzyme (representing a 50-fold molar excess over the enzyme concentration) (Fig 4). These included compounds: 10-[2-[bis(2-hydroxyethyl)amino]ethyl]-7,8-diethylbenzo[γ]pteridine-2,4-dione (compound **1**; NSC-374902); 2-((8-hydroxy-6-oxo-6H-imidazo[4,5,1-de]acridin-5-yl)amino)-N,N-dimethylethanaminium (compound **2**; NSC-645808); 5-(3-(dimethylamino)propylamino)-6H-imidazo[4,5,1-de]acridin-6-one (compound **3**; NSC-645836); 5-(2-(dimethylamino)ethylamino)-1-ethyl-6H-imidazo[4,5,1-de]acridin-6-one (compound **4**; NSC-645835); and 5-{[2-(diethylamino)ethyl]amino}-6H-imidazo[4,5,1-de]acridin-6-one hydrochloride (1:1) (compound **5**; NSC-645831). 4-(dimethylamino)-3,10,11,12a-tetrahydroxy-6-methyl-1,12-dioxo-3,4,4a,5-tetrahydro-2H-tetracene-2-carboxamide (compound **6**; NSC-118670) had an IC_50_ value of approximately 35 μM (Fig 4). Compounds **2** through **5** all contained the common acridinone (Fig 3) chemical scaffold identified during the chemical similarity mapping after composite docking (Group 4). Eight out of the ten tested compounds from this group inhibited the collagenase activity of CatK at 100 μM or lower (S2 Table).

**Fig 5:**
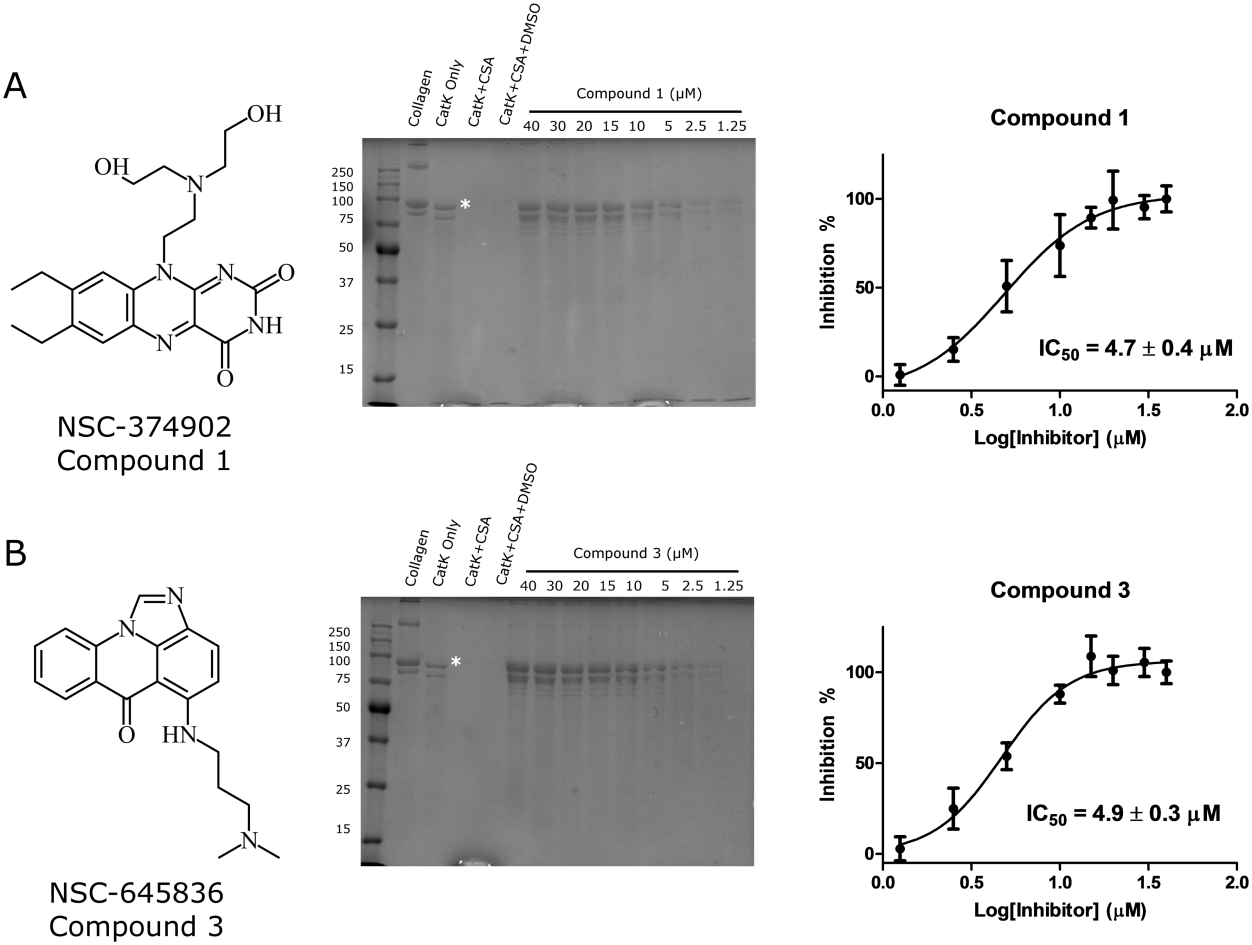
Collagenase inhibitory activity of compounds 1 and 3. The two most potent compounds, **1** (A) and **3** (B), identified through molecular docking are shown with their respective structures. Collagenase inhibitory activities with representative collagenase degradation gels are depicted with the corresponding IC_50_ curves determined from three separate experiments (n = 3). The IC_50_ values for the inhibition of collagenase activity of CatK were 4.7 ± 0.4 μM and 4.9 ± 0.3 μM for compounds **1** and **3**, respectively. * represents the α1 type I collagen peptide used to quantify the collagenase activity of CatK.

The most potent compounds identified from the druggable library subset were compounds **1** and **3**, which had IC_50_ values of 4.7 ± 0.4 μM and 4.9 ± 0.3 μM, respectively (representing a ~12-fold molar excess over the enzyme concentration) (Fig 5).

To exclude an active site inhibition for the top rated compounds, their inhibitory activity on the cleavage of Z-Phe-Arg-MCA and gelatin was evaluated. In these inhibition assays, a 5,000-fold molar excess of the inhibitor over the enzyme concentration was used. None of the five compounds with IC_50_-values of less than 20 μM in collagenase inhibition displayed inhibitory activity towards gelatin degradation at 100 μM. Some compounds revealed a minor inhibition of Z-Phe-Arg-MCA hydrolysis of up to 10% (Fig 4) at high excess inhibitor concentrations. These results suggest that the collagenase inhibitory activity observed is not a result of active site inhibition of CatK.

### Proposed binding of compounds 1 and 3

**Fig 6:**
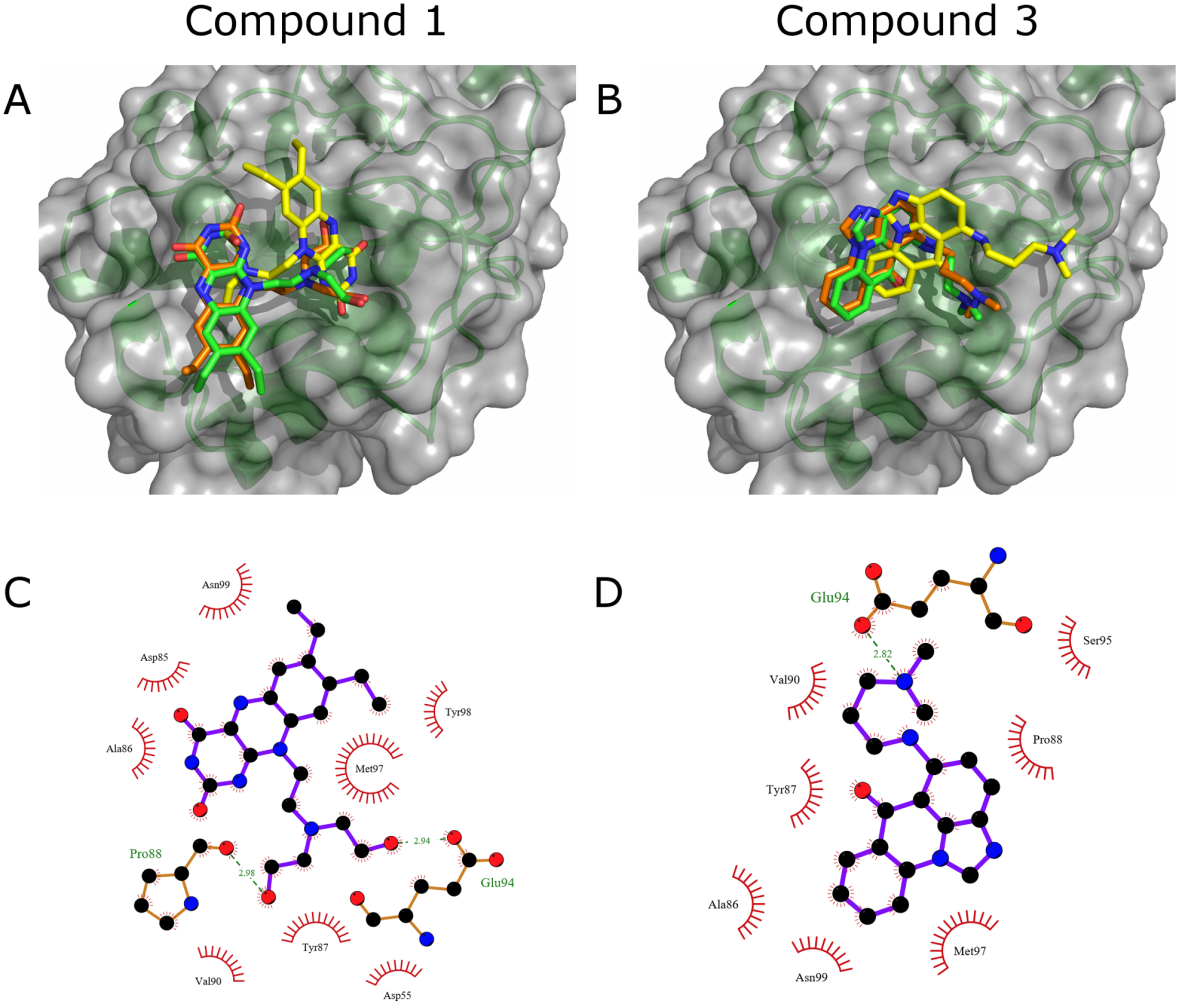
Top binding poses of compounds 1 and 3 from composite docking. Top binding poses of the most potent collagenase inhibitors, **1 (A)** and **3 (B)**, as docked using the three docking methods. The poses are depicted using sticks and colored orange (Glide), green (Surflex), and yellow (GOLD). Ligplot diagrams depicting the predicted binding of compounds **1 (C)** and **3 (D)** into ectosteric site 1 using the best binding pose calculated from Glide. Hydrogen bonds with the binding site residues are highlighted in green with the respective distances and hydrophobic interactions are shown with red dashes.

To understand the binding modes of the most potent anti-collagenase inhibitors, **1** and **3**, their binding to ectosteric site 1 was reevaluated using three different protocols with higher precision parameters and increased rotational sampling as outlined in the Methods section. The overall best binding poses from each docking algorithm are shown in Fig 6. The poses for compound **3** are all accommodated in the cleft of ectosteric site 1 with the aromatic rings of the compound making favorable hydrophobic interactions with the residues in the area. The strongest of these interactions include those with residues Ala86, Tyr87, and Pro88. In addition to the hydrophobic interactions, the compound also forms electrostatic interactions with residues Asn99 and Met97 and forms a hydrogen bond with Glu94. For compound **1**, similar favorable hydrophobic interactions can be seen with residues Ala86, Met97, and Asn99. It also forms strong electrostatic interactions with the residues in the loop including hydrogen bonding with Pro88 and Glu94. Both of the compounds had high scoring poses with theoretical *K*_i_ in the range of 5–10 μM determined from the Glide and Surflex scores, which corroborates with their experimental *in vitro* IC_50_ values. The best binding mode predicted by the GOLD algorithm for both compounds **1** and **3** were in a different conformation than those predicted by Glide and Surflex as shown in Fig 5 and showed strong hydrophobic interactions with the protein (S1 Fig). Nonetheless, the reevaluated empirical CHEMPLP scores for both compounds showed strong binding and were among the top 3% of the scores observed for the entire druggable library set.

As predicted by the Sitemap evaluation of ectosteric site 1, both compounds contained a hydrophobic core which interacted with the hydrophobic center of ectosteric site 1 (Tyr87) and contained multiple functional groups which could interact with the hydrogen-bond donating side chains in the area. Compound 3 shares a chemical scaffold with 12 other compounds found in Group 4 (Fig 3 and S1 Table), which were identified from the set of druggable compounds that also scored highly in the composite docking approach and which displayed an anti-collagenase activity.

### Complete library screening using composite docking method

Based on the results using the 14,000-member compound sub-library, we proceeded to complete the docking and testing of the entire NCI-DTP repository consisting of a total of 281,987 compounds. As in the sub-library study, the compounds were prepared with LigPrep using the previous parameters with conformers limited to three per ligand due to the increased number of compounds. The binding site definitions and docking methods were the same as used in the subset library study for each docking method. A cutoff of 4% was used for the selection of hits, which limited it to 314 compounds for further selection. The 14,000 compounds in the sub-library were also rescreened as a part of the complete library. Of the 99 compounds identified from the composite hits, only two compounds were found in the 314 compounds identified in the complete library at the 4% cutoff but were not among those with anti-collagenase activity. However, all six collagenase inhibitors identified in the subset library listed in Table 1 were found in the top 10%, representing a total of 839 compounds, of the composite hits identified in the complete library. The highest rated compound, **5**, was ranked at the 422^nd^ position. Compounds **1** and **3** ranked 803^rd^ and 609^th^, respectively.

**Table 1:** Comparison of the hit rates of each individual docking method and composite docking methods.

Docking Method	Glide	Surflex	GOLD	Total from Individual Methods	Composite Docking (Druggable)	Composite Docking (Complete)
**Top Compounds Tested**	**25**	**25**	**25**	**75**	**80**	**80**
**Active at 100 μM**	**3**	**3**	**4**	**10**	**16**	**12**
**Active at 50 μM**	**1**	**0**	**2**	**3**	**12**	**8**
**IC**_**50**_ **below 20 μM**	**0**	**0**	**1**	**1**	**5**	**3**
**IC**_**50**_ **below 5 μM**	**0**	**0**	**0**	**0**	**2**	**0**

Due to the large chemical diversity of the compounds identified, a chemical similarity map did not yield particular scaffolds of interest. However, some of the compounds identified may still be of interest for further optimization and development for higher potency. Most of the compounds identified had higher molecular weights (>700 Da), possibly as a result of the inherent bias for larger molecules in interaction scoring due to an increased number of interaction atoms [31]. The average molecular weight of the hits identified was 791 ± 281 Da, and was significantly higher than the average of 316 ± 72 Da from the screen using the initial subset of compounds (S2 Fig). The compounds were then given a composite rank as the average of their individual ranks and were also screened visually by their binding pose as in the preliminary study. Higher priority was given to compounds with extensive hydrogen bonding and hydrophobic interactions with ectosteric site 1. Compounds that primarily interacted outside of ectosteric site 1 were given less consideration. Compounds that were unavailable from the NCI/DTP repository were skipped. This led to a selection of 80 compounds for testing in *in vitro* assays.

The compounds were first screened in the collagenase assay with 12 compounds displaying inhibitory activities at 100 μM; those compounds were further evaluated at lower concentrations. Three of these compounds displayed IC_50_ values below 20 μM: dexamethasone acetate (compound **7**; NSC-39471), N-[3-(4,5-dihydro-1H-imidazol-2-yl)phenyl]-3-[[3-[[3-(4,5-dihydro-1H-imidazol-2-yl) phenyl]carbamoyl]phenyl]carbamoylamino]benzamide hydrochloride (compound **8**; NSC-53298), and 1-[2-[3-(diethylaminomethyl)-4-hydroxyphenyl]hydrazinyl]-5-[(2E)-2-[3-(diethylaminomethyl)-4-oxocyclohexa-2,5-dien-1-ylidene]hydrazinyl]naphthalene-2,6-dione hydrochloride (compound **9**; NSC-359463). The most potent compound, compound **9**, had an IC_50_-value of 8.5 μM (Table 1). In contrast with the sub-library screen, no compounds with IC_50_ values <5 μM were identified with the complete library. To test the potential active site inhibition of each of these compounds, their ability to inhibit the cleavage of non-ectosteric site-dependent substrates, gelatin and Z-Phe-Arg-MCA was tested at a 5,000-fold molar excess over the enzyme concentration. All three compounds (**7**, **8**, **9**) displayed low (<10%) active site-directed inhibition (Table 1). Similar to the previous compounds identified, the inhibition of the collagenase activity is thus likely caused by the disruption of the ectosteric site interactions and not by active site inhibition.

### Screening for aggregation and off-target inhibition for active compounds

To test whether the inhibition observed for the identified compounds are due to non-specific interactions such as compound aggregation, we tested the compounds under detergent conditions as well as their activities towards two other proteases, trypsin and matrix-metalloproteinase-1 (MMP-1). We first tested the collagenase inhibition activity of the compounds shown in Fig 4 in the presence of detergent, Triton X-100. Two detergent concentrations, were tested (0.001% and 0.005% (v/v)). Higher concentrations of Triton X-100 inhibited the collagenase reaction. At both tested concentrations, all nine compounds retained their collagenase inhibitory activities at 10 μM. Quantification of the degradation in the presence of detergent at 10 μM inhibitor concentration showed no significant difference to that observed without detergent (S3A and S3B Fig). Moreover, the IC_50_ values of compounds **1** and **3** were determined in the presence of detergent were not significantly different than those characterized without detergent (S4 Fig).

To further rule out non-specific inhibition, we tested the inhibitors with two unrelated proteases, with the serine protease, trypsin, and with matrix metalloproteinase, MMP-1. None of the compounds inhibited the cleavage of the macromolecular substrate gelatin trypsin at 50 μM concentrations (S3C Fig). Likewise, the degradation of the fluorogenic peptide, Z-Phe-Arg-MCA by trypsin was not significantly inhibited by the compounds (<10%) with the exception of compound **6**, which showed 60% inhibition at 50 μM (5,000-times molar excess). Compounds **1** and **3** were further evaluated for their inhibition of MMP-1. Both compounds showed no inhibitory activity at 50 μM (approximately 10-times their IC_50_ concentrations for the collagenase activity of CatK) towards the collagenase activity of MMP-1. Additionally, both compounds showed no inhibitory activity at 50 μM (5,000 times excess enzyme concentrations) for the gelatinase activity of MMP-1 (S3D Fig).

### Comparison of composite docking method with individual methods

In order to evaluate which method is more efficient at identifying ectosteric inhibitors, we compared the composite docking method with the individual docking methods for the identification of active compounds from the NCI-DTP Repository. We evaluated the top 25 available compounds from each individual docking method for their *in vitro* inhibitory activities towards collagen, gelatin, and Z-Phe-Arg-MCA degradation and compared the results with those obtained from the composite method.

For Surflex identified ligands, only three compounds displayed inhibitory activity at 100 μM in the collagenase assay (S3 Table). However, none of these compounds were active at 50 μM. None of the top 25 compounds identified by Surflex alone were found among the composite hit list. From the top 25 compounds identified in the Glide screen, three compounds showed inhibitory activity for the collagenase activity of CatK at 100 μM and one compound was active at 50 μM (S3 Table). Of the top 25 compounds identified from Glide alone, only two were identified by the composite method, but these compounds did not display any significant collagenase inhibitory activity. Finally, the top 25 compounds identified with GOLD showed four compounds with inhibitory activity at 100 μM and two compounds effective at 50 μM (S3 Table). This included compound **9**, which had been identified during composite docking and had an IC_50_ value of 8.5 μM in the collagenase assay.

Compared with the composite docking method, the individual methods were less effective at identifying potent compounds of the collagenase activity in CatK (Table 1). *In vitro* testing of 75 compounds in total from the three individual methods only yielded 10 compounds with inhibitory effect at 100 μM. The most potent compound was **9**, which was identified with GOLD, with an IC_50_ value of 8.5 μM. In comparison, the composite docking method screening the entire library yielded 12 compounds active at 100 μM, with three compounds (**7**, **8**, **9**) having IC_50_ values below 20 μM from 80 tested. Composite docking using the sub-library had the highest hit rate with 16 compounds showing inhibition at 100 μM. Five compounds (**1, 2, 3, 4, 5**) had IC_50_ values below 20 μM and two (**1, 3**) of which had IC_50_ values of approximately 5 μM from the 80 compounds tested from the composite screen (Table 1).

### Osteoclast-bone resorption assays using most potent compounds identified from NCI-DTP repository

**Fig 7:**
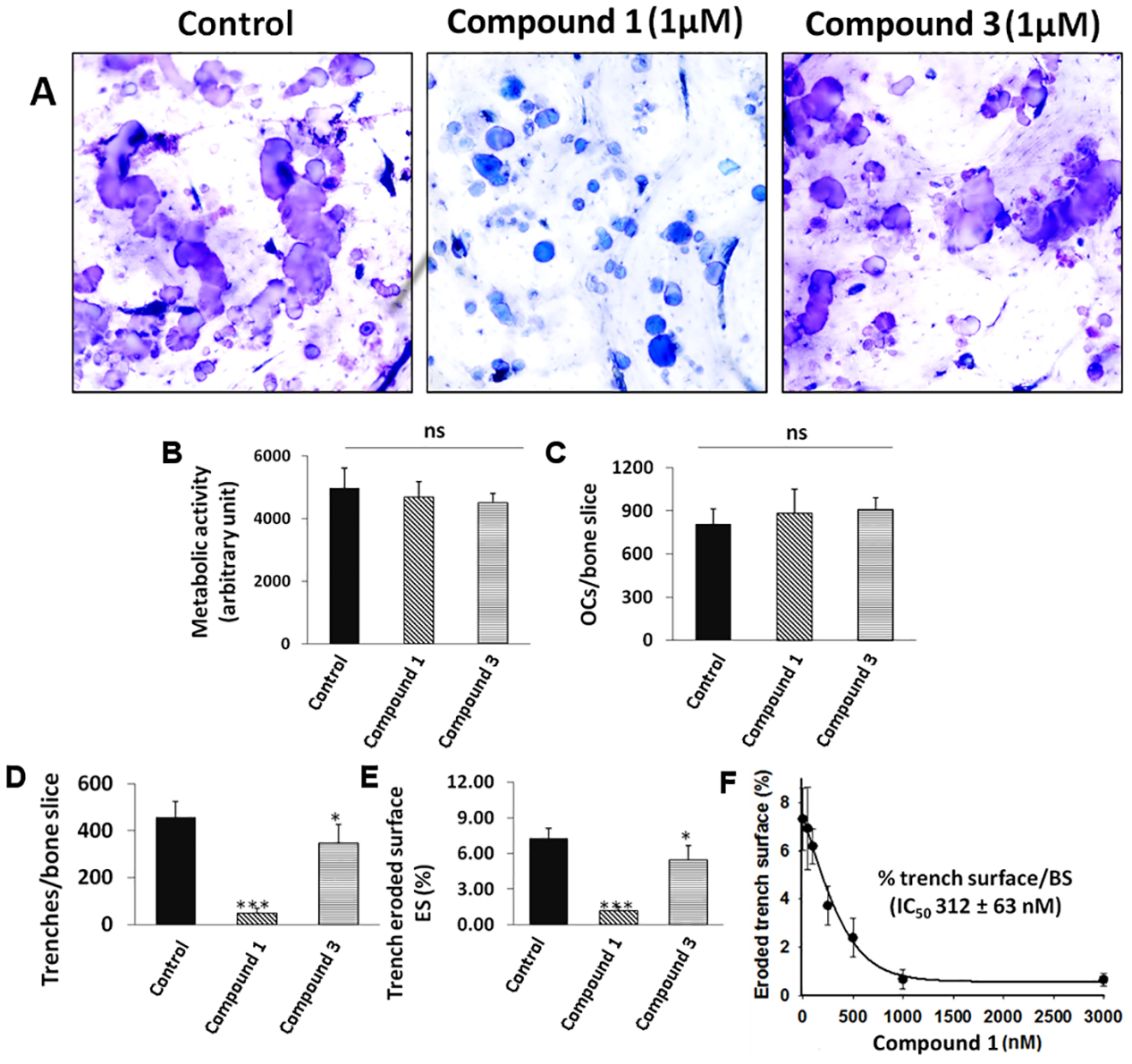
The effects of NSC-374902 and NSC-645836 on human osteoclasts and bone resorption activity. **(A)** Representative images of osteoclast-generated resorption cavities in the presence or absence of compounds **1** and **3**. Large meandering cavities known as trenches can be observed and represent collagen degradation event. Small round cavities are demineralized pit area with no or little collagen degradation. Mature human osteoclasts were cultured on bovine bone slices for 72 hours in the absence or presence of inhibitors (1μM). **(B)** Metabolic activity and **(C)** number of osteoclasts after treatment with NSC compounds compared with untreated cells show no significant differences. **(D)** Number of trenches and **(E)** percentage of eroded surface area under untreated and inhibitor treated (1 μM) conditions show significant reduction for compound **1** (p<0.001) and **3** (p<0.05). **(F)** The IC_50_ value of compound **1** for the inhibition of trench eroded surface per bone surface was 312 ± 63 nM. The IC_50_ value was determined from three independent experiments where 5 bone slices in each condition were analyzed. Data represent mean ± SD. ‘ns’, not significant; * p< 0.05; ***p< 0.001.

The two most potent compounds identified in the collagenase assays were tested in cell-based osteoclast bone resorption assays to evaluate their ability to inhibit the degradation of bone. Fig 7A shows toluidine-stained osteoclast-mediated resorption events on bone surfaces in the absence or presence compounds **1** and **3** using human osteoclasts. In untreated cultures, long deep trenches with small round pits were present, indicating extensive bone resorption. Compound **1**-treated cultures (1 μM) showed mostly small round demineralized pits indicating an almost complete inhibition of CatK-mediated bone resorption. Round small resorption pits represent CatK-independent demineralization events. Quantification of the number of osteoclasts and metabolic activity showed no significant changes between inhibitor-treated and untreated samples, suggesting no toxicity at the tested inhibitor concentrations (Fig 7B and 7C). Quantitative analysis of the resorption parameters revealed that 1 μM of compound **1** was very effective in reducing the total eroded surface by osteoclasts. The number of trenches and total trench-eroded surface was significantly less than in the untreated sample (p<0.005) (Fig 7D and 7E). The IC_50_ value for the inhibitory effect on the total eroded surface/bone surface for compound **1** was 312 ± 63 nM (Fig 7F). For comparison, the active site-directed inhibitor, odanacatib, had an IC_50_ value for human-osteoclast-mediated bone resorption of 14.6 nM [17]. However, odanacatib as most other active site-directed inhibitors showed significant side effects leading to the termination of the further development of active site-directed CatK inhibitors [14].

Surprisingly, compound **3** had a lesser effect on trench formation (Fig 7A, 7D and 7E). The compound was less effective in reducing bone resorption; however the total eroded surface and number of trenches was still significantly reduced when compared to the untreated samples (p<0.01). The solubility and the ability to cross the cell membrane may play a role in the reduced potency. Both compounds had octanol/water partition coefficients (cLogP) in the druggable range (-0.4 to 5.6) but compound **3** (3.71) had a higher value than compound **1** (0.60). This indicates a lower water solubility, which may have played a role in its lower cell efficiency [32]. Despite the lower potency, the scaffold of this compound may still be used to further refine potential collagenase inhibitors of CatK.

## Discussion

Molecular docking approaches have been shown to aid in identifying potential chemical scaffolds for novel targets [33]. Several computational methods are available to find potential ligands for a druggable target. These include library docking, fragment-based synthesis, as well as molecular dynamics simulations [34, 35]. Computational approaches are faster and more cost-effective when compared with traditional high-throughput experimental screens.

Here, we employed various molecular docking methods to identify non-active site-directed inhibitors of CatK. CatK has been previously shown to be an attractive target for the treatment of osteoporosis. Multiple active site-directed CatK inhibitors have been evaluated in past clinical trials of osteoporosis. Despite superior efficacy in increasing bone mineral density and reducing fracture rates all compounds have failed because of side effects, including cardiovascular complications and skin fibrosis [13, 14]. The exact mechanisms of these side effects remain unknown but may be due to the inhibition of CatK-mediated degradation of regulatory proteins such as TGF-ß1. We have previously shown that inhibitors targeting the ectosteric site 1 specifically blocks osteoclast bone resorption *in vitro* and *in vivo* without disrupting its active site activities [17]. Therefore, we aimed at identifying novel drug scaffolds which target the ectosteric site I of the protease. Sitemap characterization of this binding site shows druggability and many interacting residues for compounds to bind favorably.

Using a combination of three different docking algorithms, we identified several ectosteric site-based inhibitors of CatK. Most screens use a maximum of two different docking methods [36–38], which may account for the high false positive rate [39]. Our composite approach was up to five-fold more effective at identifying potent collagenase inhibitors (IC_50_ <20 μM) when compared to the single docking methods and was able to identify two effective inhibitors with IC_50_ values of below 5 μM (Fig 4). By filtering the library to drug-like compounds using the Lipinski rule of five and bioassay activities, we were able to further increase the hit rates and potency of identified compounds. This difference in potency and hit rates might be due to the difficulty in estimating accurate binding affinities for larger compounds with multiple interacting atoms and the general bias for interaction scoring for compounds with higher molecular weights [40, 41]. Starting with a smaller library of low molecular weight compounds and employing multiple scoring strategies may help in increasing both the potency and hit rate of identifying compounds and decrease the number of false positives. Through structural similarity mapping of the composite docking hits, we were able to distinguish several novel chemical scaffolds with a selective collagenase inhibitory activity.

Examination of the best predicted binding poses of the two most potent compounds, **1** and **3**, revealed favorable interactions with the residues in the pocket of ectosteric site 1. Both of the compounds contained a hydrophobic ring system, acridinone or pteridine for compounds **3** and **1**, respectively, and functional groups, which can participate in non-covalent interactions with the other residues identified in the binding site. The poses and interactions calculated by the respective algorithms were similar and matched closely with the potential interactions predicted in the Sitemap analysis. Despite similar potency in collagenase inhibition, there was a noted difference in their efficacy in the cell-based resorption assays. Compound **1** was significantly more potent in preventing bone resorption than compound **3**. Their calculated membrane permeabilities (nm/s) (2.4 for compound **1** and 1.5 for compound **3**) determined from the calculated physicochemical properties and cLogP values (Schrödinger Inc.) may have played a role in the differences in potency observed for the osteoclast-based assays [42].

We also investigated whether the presence of assay interference such as non-specific binding due to small molecule aggregates could generate false positives in our screening assays [43]. Detergents break up aggregation and thus would reduce potential aggregation-based inhibition [44]. All nine compounds in Fig 4 retained their anti-collagenase activities in the presence of Triton X-100 at two concentrations (0.001% and 0.005% v/v). Triton X-100 had no effect on the IC_50_ values of the two most potent compounds (**1** and **3**) (S4 Fig).

In order to exclude off-target effects of the identified CatK ectosteric inhibitors, we evaluated the potential inhibitory effect on trypsin and MMP-1. All nine compounds in Fig 4 did not show any inhibitory activity towards the cleavage of gelatin by trypsin at their highest inhibitor concentrations (50 μM) (S3C Fig). Moreover, compounds **1** and **3** did not show inhibitory activity towards MMP-1 for the degradation of collagen or gelatin (S3D Fig). Taken together, this indicates that the identified compounds are selective CatK inhibitors, which inhibit specifically the collagenase activity of the protease.

The IC_50_ value for compound **1** in the osteoclast resorption assay was 312 nM. This is close to the IC_50_ value of tanshinone IIA sulfonate (240 nM), which showed efficacy in ovariectomiced mice [18]. The overall potency is still about 15–20 times lower than for the most potent active site-directed CatK inhibitor, odanacatib [17] but may have the advantage of avoiding side effects seen with odanacatib and other CatK inhibitors [13, 45]. Compound **1** may serve as a scaffold for the optimization of more potent CatK collagen specific inhibitors.

It should be noted that some of the compounds identified in our screening process have shown bioactivities in other assays. Compounds **1** and **9** in particular, were previously shown to be active in an NCI Yeast Anticancer Drug Screen [46]. Compound **3** was also shown to be active in an inhibitor screen of a quinone oxidoreductase and a structurally related compound has been shown to be a potent inhibitor of the enzyme and may have chemo-protectant potential [47].

In conclusion, we have demonstrated that *in silico* molecular docking methods can be successfully employed to identify ectosteric inhibitors of CatK. The hit rate was substantially increased when a composite docking approach was employed. We believe that this method can be applied to other targets as well and can be effective for targets where a high-throughput assay has not been developed or is not cost-effective.

## Supporting information

S1 Table Summary of scaffolds identified through composite docking using druggable compounds from the NCI/DTP repository. (DOCX)

S2 Table Summary of collagenase inhibitors at 100 μM identified through composite docking using druggable compounds from the NCI/DTP repository. (DOCX)

S3 Table Summary of collagenase inhibitors active at 100 μM identified with individual docking methods from the complete NCI/DTP repository. (DOCX)

S1 Fig LigPlot diagrams of top binding poses of compounds 1 (A) and 3 (B) using GOLD. Ligplot diagrams of the top binding poses of compounds 1 and 3 using GOLD show strong interactions with the protein.
(PNG)

S2 Fig Distribution of molecular weights of composite docking hits identified from the NCI/DTP repository. The frequency distribution data of the hits identified through composite docking shows a higher average molecular weight for the complete library (791 ± 281 Da) than the druggable subset (316 ± 72 Da).
(PNG)

S3 Fig Effect of Triton X-100 on the collagenase activity of compounds 1–9 and lack of off-target inhibition. The collagen inhibition activity of compounds 1–9 was not affected by 0.005% (A) Triton X-100 shown with the corresponding representative SDS-PAGE gel. Identical results were obtained in the presence of 0.001% Triton X-100. (Data not shown.) (B) Quantification of the α1 type I bands (*) from three separate experiments (n = 3) showed no significant effect of the detergent on collagen degradation inhibition. Compounds 1–9 also did not show off-target inhibition of trypsin-mediated digestion of gelatin (C) at 50 μM in the presence of 10 nM enzyme. (D) Compounds 1 and 3 did not show inhibition of MMP-1-mediated degradation of collagen and gelatin at 50 μM inhibitor concentrations. 400 nM and 10 nM MMP-1 was used for collagen and gelatin degradation, respectively. Representative SDS-PAGE gels for the degradation experiments are shown.
(PNG)

S4 Fig IC_50_ curves of the collagenase activity of compounds 1 (A) and 3 (B) with 0.005% Triton X-100. The IC_50_ values of the collagenase inhibitory activity of compounds 1 and 3 were 5.1 ± 1.0 μM and 5.2 ± 1.5 μM, respectively.
(TIF)

## Abbreviations

CatK: cathepsin K
CTx-I: carboxy-terminal telopeptide of type I collagen
DTP: Developmental Therapeutics Program
DTT: dithiothreitol
E64: L-3-carboxy-*trans*-2–3-epoxypropionyl-leucylamido-(4-guanidino)-butane
EDTA: ethylenediametetraacetate
NCI: National Cancer Institute
Z-FR-MCA: Benzyloxycarbonyl-Phe-Arg-4-methyl-coumarin-7-amide
