# Supplementary material for: A composite docking approach for the identification and characterization of ectosteric inhibitors of cathepsin K
Source: PLoS One. 2017 Oct 31;12(10):e0186869. doi: 10.1371/journal.pone.0186869 (PMC5663397; doi:10.1371/journal.pone.0186869)
Supplement: S1 Table — (DOCX) [file pone.0186869.s001.docx]

**S1 Table. Summary of scaffolds identified through composite docking using druggable compounds from the NCI/DTP Repository listed by NSC number**

| **Group 1** | | | | | | | | | |
| --- | --- | --- | --- | --- | --- | --- | --- | --- | --- |
|   **642740** | |   **623611** | |   **642890** | | | |   **647595** | |
| **Group 2** | | | | | | | | | |
| ****  **688955** | | | ****  **664938** | | | | ****  **102816** | | |
| ****  **651036** | | | | | | ****  **690204** | | | |
| **Group 3** | | | | | | | | | |
| **147786** | | |   **76356** | | | | **145386** | | |
| **Group 4** | | | | | | | | | |
| ****  **645808** | | | **645811** | | | | **645812** | | |
| **645815** | | | **645816** | | | | ****  **645821** | | |
| ****  **645823** | | | ****  **645824** | | | | ****  **645825** | | |
| ****  **645831** | ****  **645833** | | | | **645835** | | | | ****  **645836** |
