# Supplementary material for: A composite docking approach for the identification and characterization of ectosteric inhibitors of cathepsin K
Source: PLoS One. 2017 Oct 31;12(10):e0186869. doi: 10.1371/journal.pone.0186869 (PMC5663397; doi:10.1371/journal.pone.0186869)
Supplement: S2 Table — (DOCX) [file pone.0186869.s002.docx]

**S2 Table. Summary of collaganase inhibitors at 100 µM identified through composite docking using druggable compounds from the NCI/DTP Repository**

| **Structures of Collagenase Inhibitors Identified Through Composite Docking Listed by NSC Number** | | | | | |
| --- | --- | --- | --- | --- | --- |
|   **374902** | | **645808** | | **645831** | |
| ****  **645835** | | ****  **645836** | | ****  **118670** | |
|   **124845** | |   **611243** | |   **54055** | |
|   **60472** | |   **62378** | |   **76356** | |
|   **79058** |   **81540** | |   **106540** | |   **108608** |
