# Supplementary material for: A composite docking approach for the identification and characterization of ectosteric inhibitors of cathepsin K
Source: PLoS One. 2017 Oct 31;12(10):e0186869. doi: 10.1371/journal.pone.0186869 (PMC5663397; doi:10.1371/journal.pone.0186869)
Supplement: S3 Table — (DOCX) [file pone.0186869.s003.docx]

**S3 Table. Summary of collagenase inhibitors active at 100 µM identified with individual docking methods from the complete NCI/DTP Repository**

| **Surflex Only Identified Compounds** | |
| --- | --- |
|   **42322** |   **645812** |
|   **80975** | |

| **Glide Only Identified Compounds** | |
| --- | --- |
|   **80116 (Collagenase IC_50_: 89 ± 9.5 μM)** |   **136985** |
|   **85206** | |

| **GOLD Only Identified Compounds** | |
| --- | --- |
|   **53213** |   **70530** |
|   **719315 (Collagenase IC_50_: 88 ± 6.3 μM)** | |
