# Supplementary figures and images for: A composite docking approach for the identification and characterization of ectosteric inhibitors of cathepsin K
Source: PLoS One. 2017 Oct 31;12(10):e0186869. doi: 10.1371/journal.pone.0186869 (PMC5663397; doi:10.1371/journal.pone.0186869)

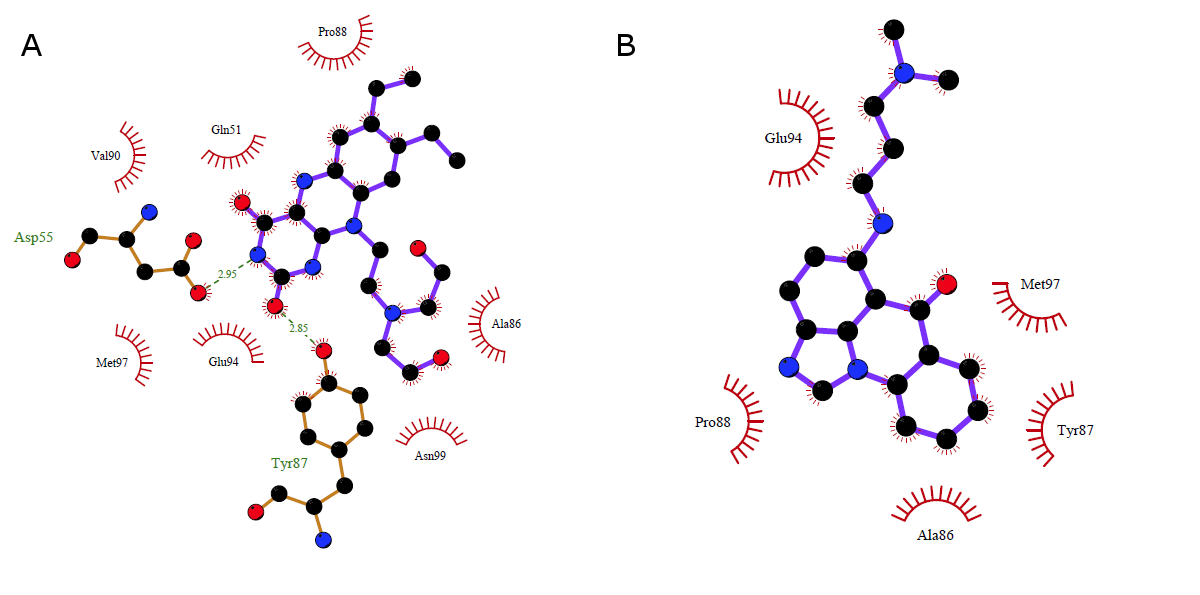

Supplement: S1 Fig — Ligplot diagrams of the top binding poses of compounds 1 and 3 using GOLD show strong interactions with the protein. (PNG) [file pone.0186869.s004.png]

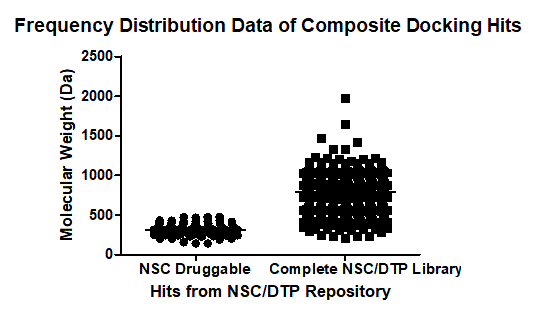

Supplement: S2 Fig — The frequency distribution data of the hits identified through composite docking shows a higher average molecular weight for the complete library (791 ± 281 Da) than the druggable subset (316 ± 72 Da). (PNG) [file pone.0186869.s005.png]

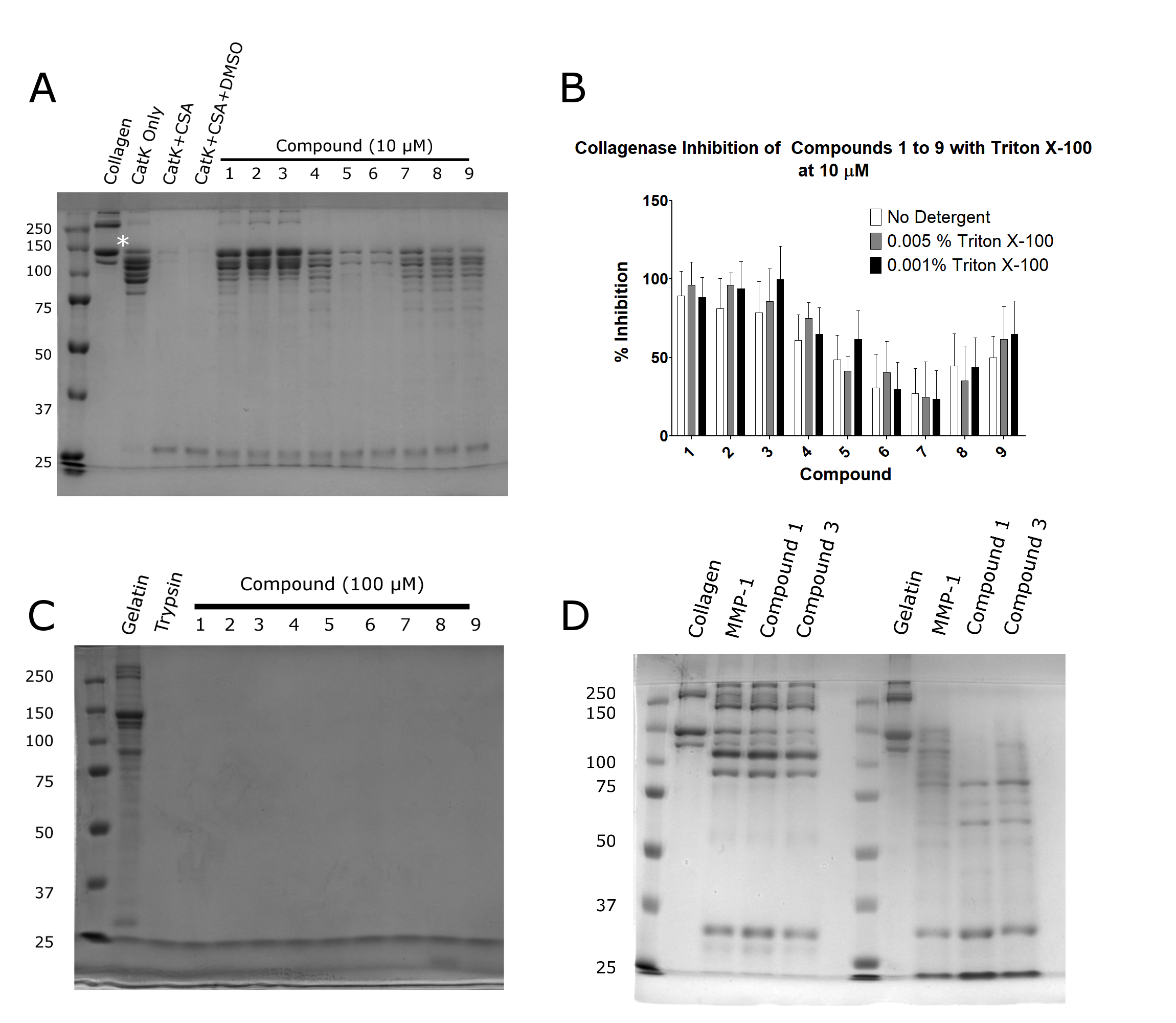

Supplement: S3 Fig — The collagen inhibition activity of compounds 1–9 was not affected by 0.005% (A) Triton X-100 shown with the corresponding representative SDS-PAGE gel. Identical results were obtained in the presence of 0.001% Triton X-100. (Data not shown.) (B) Quantification of the α1 type I bands (*) from three separate experiments (n = 3) showed no significant effect of the detergent on collagen degradation inhibition. Compounds 1–9 also did not show off-target inhibition of trypsin-mediated digestion of gelatin (C) at 50 μM in the presence of 10 nM enzyme. (D) Compounds 1 and 3 did not show inhibition of MMP-1-mediated degradation of collagen and gelatin at 50 μM inhibitor concentrations. 400 nM and 10 nM MMP-1 was used for collagen and gelatin degradation, respectively. Representative SDS-PAGE gels for the degradation experiments are shown. (PNG) [file pone.0186869.s006.png]

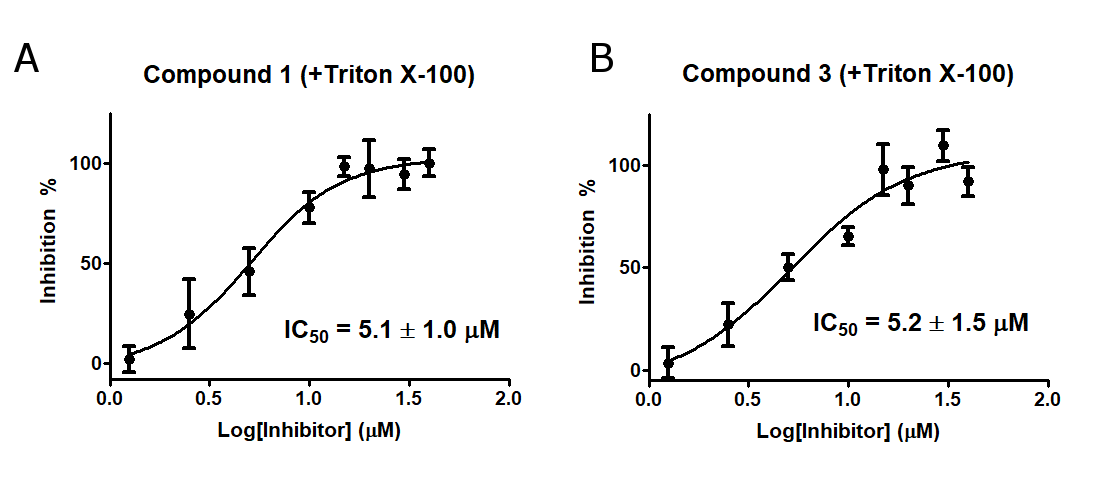

Supplement: S4 Fig — The IC50 values of the collagenase inhibitory activity of compounds 1 and 3 were 5.1 ± 1.0 μM and 5.2 ± 1.5 μM, respectively. (TIF) [file pone.0186869.s007.tif]
